# Magnetic Resonance Features of Liver Mucinous Colorectal Metastases: What the Radiologist Should Know

**DOI:** 10.3390/jcm11082221

**Published:** 2022-04-15

**Authors:** Vincenza Granata, Roberta Fusco, Federica De Muzio, Carmen Cutolo, Sergio Venanzio Setola, Federica Dell’Aversana, Andrea Belli, Carmela Romano, Alessandro Ottaiano, Guglielmo Nasti, Antonio Avallone, Vittorio Miele, Fabiana Tatangelo, Antonella Petrillo, Francesco Izzo

**Affiliations:** 1 Division of Radiology, Istituto Nazionale Tumori IRCCS Fondazione Pascale—IRCCS di Napoli, 80131 Naples, Italy; s.setola@istitutotumori.na.it (S.V.S.); a.petrillo@istitutotumori.na.it (A.P.); 2 Medical Oncology Division, Igea SpA, 80013 Naples, Italy; r.fusco@igeamedical.com; 3 Department of Medicine and Health Sciences “V. Tiberio”, University of Molise, 86100 Campobasso, Italy; demuziofederica@gmail.com; 4 Department of Medicine, Surgery and Dentistry, University of Salerno, 84084 Salerno, Italy; carmencutolo@hotmail.it; 5 Division of Radiology, Università Degli Studi Della Campania Luigi Vanvitelli, 80138 Naples, Italy; federica.dellaversana@unicampania.it; 6 Division of Epatobiliary Surgical Oncology, Istituto Nazionale Tumori IRCCS Fondazione Pascale—IRCCS di Napoli, 80131 Naples, Italy; a.belli@istitutotumori.na.it (A.B.); f.izzo@istitutotumori.na.it (F.I.); 7 Division of Abdominal Oncology, Istituto Nazionale Tumori IRCCS Fondazione Pascale—IRCCS di Napoli, 80131 Naples, Italy; c.romano@istitutotumori.na.it (C.R.); a.ottaiano@istitutotumori.na.it (A.O.); g.nasti@istitutotumori.na.it (G.N.); a.avallone@istitutotumori.na.it (A.A.); 8 Italian Society of Medical and Interventional Radiology (SIRM), SIRM Foundation, via della Signora 2, 20122 Milan, Italy; vmiele@sirm.org; 9 Division of Radiology, Azienda Ospedaliera Universitaria Careggi, 50134 Florence, Italy; 10 Division of Pathological Anatomy and Cytopathology, Istituto Nazionale Tumori IRCCS Fondazione Pascale—IRCCS di Napoli, 80131 Naples, Italy; f.tatangelo@istitutotumori.na.it

**Keywords:** MRI, mucinous liver metastases, LI-RADS

## Abstract

Purpose: The aim of this study is to assess MRI features of mucinous liver metastases compared to non-mucinous metastases and hepatic hemangioma. Methods: A radiological archive was assessed from January 2017 to June 2021 to select patients subjected to liver resection for CRCLM and MRI in the staging phase. We selected 20 patients with hepatic hemangioma (study group B). We evaluated (a) the maximum diameter of the lesions, in millimeters, on T1-W flash 2D in phase and out phase, on axial HASTE T2-W and on portal phase axial VIBE T1 W; and (b) the signal intensity (SI) in T1-W sequences, in T2-W sequences, Diffusion-Weighted Imaging (DWI) sequences and apparent diffusion coefficient (ADC) maps so as to observe (c) the presence and the type of contrast enhancement during the contrast study. The chi-square test was employed to analyze differences in percentage values of the categorical variable, while the non-parametric Kruskal–Wallis test was used to test for statistically significant differences between the median values of the continuous variables. A *p*-value < 0.05 was considered statistically significant. Results: The final study population included 52 patients (33 men and 19 women) with 63 years of median age (range 37–82 years) and 157 metastases. In 35 patients, we found 118 non-mucinous type metastases (control group), and in 17 patients, we found 39 mucinous type metastases (study group A). During follow-up, recurrence occurred in 12 patients, and three exhibited mucinous types among them. In the study group, all lesions (100%) showed hypointense SI on T1-W, very high SI (similar to hepatic hemangioma) in T2-W with restricted diffusion and iso-hypointense signals in the ADC map. During the contrast study, the main significant feature is the peripheral progressive enhancement.

## 1. Introduction

Colorectal cancer (CRC) remains one of the largely diagnosed tumors worldwide [1,2,3,4,5,6,7]. Today, the main cause of poor prognosis is metastatic disease, and the liver remains the typical site of distant lesions. It has been reported that, at the diagnosis time, about 20% of patients have liver colorectal cancer metastases (CRCLM), while 40–50% of them will develop metastases during surveillance [8,9,10,11,12,13,14,15,16]. Although liver metastases surgical resection is the best chance of long-term survival, almost 60% of patients will develop local recurrence even after an R0 resection of primary liver metastases. Several recurrence risk factors have been recognized, such as T3/T4 CRC, node-positive primary tumor, synchronous liver metastases, more than three liver lesions and a positive resection margin [17,18]. The administration of adjuvant chemotherapy, with a complete or partial response, is shown to be associated with a lower recurrence risk [19].

Regarding histological subtypes, there is inadequate information on the histological subtypes’ role in CRCLM patient outcomes. The most common histological subtype of CRC is adenocarcinoma not otherwise specified (NOS), followed by mucinous adenocarcinoma, which represents 5–15% of all CRCs. Mucinous adenocarcinoma is correlated to a greater burden of KRAS and BRAF mutations, a higher rate of microsatellite instability, and a higher rate of CpG island methylator phenotype high (CIMP-H) tumors [20,21,22]. It is known that, compared to the NOS subtype, the mucinous type is correlated to a higher risk of metastases, worse overall survival (OS) and an impaired response to conventional chemotherapy [20,21,22].

In this scenario, it is clear that a proper identification and characterization of liver mucinous metastases allows for a better patient selection process, avoiding unnecessary treatment. Consequentially, the radiologist plays a crucial role in the multidisciplinary team of CRCLM patients [23,24,25,26,27,28,29]. Although computed tomography (CT) is usually the imaging tool utilized for staging and surveillance [30,31,32,33], Magnetic Resonance Imaging (MRI) is the main appreciated imaging method in liver assessment thanks to its capacity to offer conventional and functional data that improve lesion characterizations [34,35,36,37,38,39,40,41,42].

Although several researchers have assessed the MRI features of liver metastases in patients with colorectal cancer [43,44,45,46], to the best of our knowledge, few studies have evaluated mucinous features obtained by MRI studies. In the present study, we assessed the features of mucinous liver metastases compared to NOS metastases.

## 2. Materials and Methods

The Ethical Committee board of Naples National Cancer Institute admitted this retrospective study. Patient informed consent was renounced due to the study’s retrospective nature. All procedures were performed according to National Guidelines.

The radiological archive was assessed from January 2017 to June 2021 to select patients subjected to CRCLM surgical resection that have been subjected to MRI during the staging phase. The inclusion criteria were as follows: (1) pathological proven lesions; (2) MR studies with high-quality images and (3) a follow-up CT scan of at least six months after liver surgery. The exclusion criteria were as follows: (1) discordance among the radiological and the pathological diagnosis and (2) no contrast MRI studies. 

Moreover, we selected 20 consecutive patients with hepatic hemangioma.

### 2.1. MR Imaging Protocol 

MR imaging was performed using a 1.5 T MR (Magnetom Symphony, with Total Imaging Matrix Package, Siemens, Erlangen, Germany) with an 8-element body and phased array coils. The MRI study protocol included conventional sequences that are T1 weighted (W), without contrast medium administration, and T2-W, which includes Diffusion-Weighted Imaging (DWI) with seven b values to obtain functional parameters with a mono-exponential model and T1-W sequences after the administration of the contrast medium. The MR protocol was described in detail in [25,28].

According to the different phases of patient management, our study protocol includes the possibility to administrate a liver-specific contrast (in pre-surgical setting) and a non-liver-specific contrast (in the characterization and staging phase). In this study, we assessed images obtained with a non-specific agent: the Gd-BT-DO3A (Gadovist, Bayer Schering Pharma, Germany). All patients received 0.1 mL/kg of Gd-BT-DO3A by means of a power injector (Spectris Solaris^®^ EP MR, MEDRAD Inc., Indianola, IA, USA), at an infusion rate of 2 mL/s. 

The contrast study protocol includes the arterial phase (35 s delay), portal/venous phase (90 s) and equilibrium phases (120 s).

### 2.2. Images Analysis 

MR studies were assessed in consensus by three expert radiologists that were non-blinded to pathological diagnosis. All lesions were analyzed, and we evaluated the following:

1. The maximum diameter of the lesions, in millimeters, on axial T1-W sequences, on axial T2-W sequence and in the portal phase of the contrast study.
2. The signal intensity (SI) in T1 W, in T2-W, DWI sequences and the apparent diffusion coefficient (ADC) map.
3. The presence and the type of contrast enhancement (CE) during the contrast study.

Regarding point 2, the SI of the metastases was categorized as isointense, hypointense, hyperintense or with targetoid appearance (TA), with respect to the surrounding liver parenchyma. 

DWI was assessed using the mono-exponential model, as previously reported [25], and is based on manual ROI segmentation using the median value for each b value. Data analysis was performed using an in-house code written in Matlab (The MathWorks, Inc., Natick, MA, USA).

All MR features were assessed according to Liver Imaging Reporting and Data System (LI-RADS) version 2018 [v2018] [47]. 

### 2.3. Reference Standard 

Histopathologic data, including metastasectomy and biopsy results, from the routine report, were used as the reference standard for determining metastasis histological subtypes. 

### 2.4. Statistical Analysis 

A chi-square test was employed to analyze differences in percentage values of categorical variables, while the non-parametric Kruskal–Wallis test was used to test for statistically significant differences between the median values of the continuous variables.

A *p*-value < 0.05 was considered statistically significant. Statistical analysis was obtained by means of the Statistic Toolbox of Matlab (The MathWorks, Inc., Natick, MA, USA).

## 3. Results 

The final study population included 52 patients (33 men and 19 women) with 63 years of median age (range 37–82 years) and 157 metastases. 

In 35 patients, we found 118 non-mucinous type metastases (control group A), and in 17 patients, we found 39 mucinous type metastases (study group).

Twenty patients with hepatic hemangioma were categorized as control group B.

During follow-up, recurrence occurred in 12 patients, and among them, 3 were in the study group.

The characteristics of the patients are summarized in Table 1.

### 3.1. T1-W Signal Intensity

Study group: All lesions (39–100%) showed hypointense signal.

Control group A: All lesions showed hypointense signal.

Control group B: All lesions showed hypointense signal.

We found no statistically significant difference among the groups (*p* value > 0.01 with the chi-squared test).

### 3.2. T2-W Signal Intensity and Diffusion

**Figure 1:**
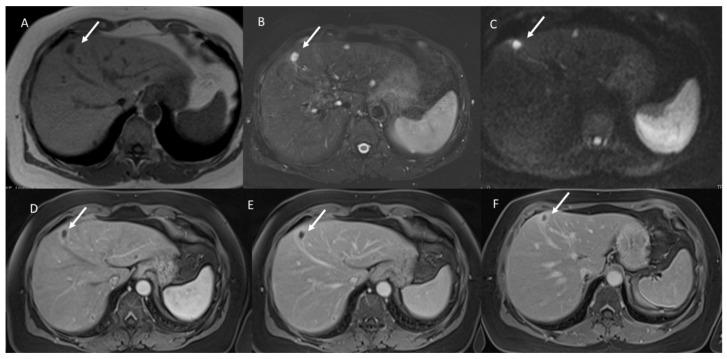
Mucinous liver metastasis on IV hepatic segment (arrow). In (**A**) T1-W image, the lesion shows hypointense signal. In (**B**), the lesion shows very high SI in T2-W and restricted diffusion in b800 s/mm^2^ (**C**). During contrast study ((**D**) arterial phase, (**E**) portal phase and (**F**) equilibrium phase), the lesion shows Rim APHE in all phases.

Study group: All lesions (39–100%) showed a very high signal (similar to hepatic hemangioma [25]) (Figure 1) with restricted diffusion and iso-hypointense signals in the ADC map.

Control group A: Among the 118 lesions, 38 (32.2%) lesions showed targetoid appearance in T2, DWI sequences and ADC map (due to central necrosis), and 80 (67.8%) lesions showed hyperintense signals in T2-W (lesser than hemangioma) and restricted diffusion with hypointense signals in the ADC map.

Control group B: All lesions showed hyperintense signal, similarly to the gallbladder, restricted diffusion and isointense signals on the ADC map.

The hyperintense signal and targetoid appearance on DWI showed a statistically significant difference between the study group and the control group A (*p*-value << 0.01 on the chi-squared test). Thus, we found a statistically significant difference between the iso-hypointense signal and the hypointense signal on the ADC map (*p*-value << 0.01 on the chi-squared test).

We found no statistically significant difference between the study group and control group B (*p*-value > 0.01 on the chi-squared test).

### 3.3. Arterial Phase Appearance

**Figure 2:**
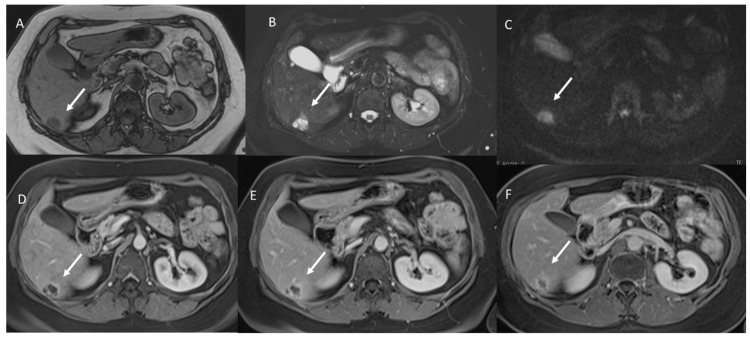
Mucinous liver metastasis (arrow) on VI hepatic segment. In (**A**) T1-W image, the lesion shows hypointense signal. In (**B**), the lesion shows very high SI in T2-W and restricted diffusion in b800 s/mm^2^ (**C**). During contrast study ((**D**) arterial phase, (**E**) portal phase and (**F**) equilibrium phase), the lesion shows Rim APHE in all phases.

Study group: All lesions showed Rim APHE (Figure 2). In this group, we found transient hepatic signal intensity differences (THID) in 28 (71.8%) lesions [48]

Control group A: All lesions showed Rim APHE. Twenty-five lesions (21.2%) showed THID.

Control group B: All lesions showed Rim APHE. No lesions showed THID.

The THID presence between the groups was statistically different (*p*-value << 0.01 on the chi-squared test).

### 3.4. Portal Phase Appearance

**Figure 3:**
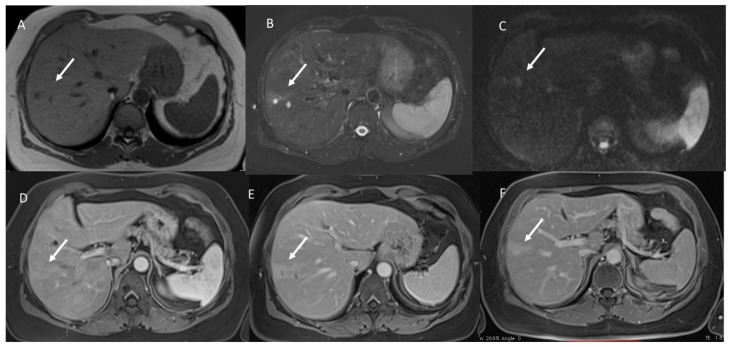
Mucinous liver metastasis (arrow) on VII-VIII hepatic segment. In (**A**) T1-W image, the lesion shows hypointense signals. In (**B**), the lesion shows very high SI in T2-W and restricted diffusion in b800 s/mm^2^ (**C**). During contrast study ((**D**) arterial phase, (**E**) portal phase and (**F**) equilibrium phase), the lesion shows progressive enhancement from arterial to delayed phase on contrast study.

Study group: All lesions showed non-peripheral washouts with targetoid appearance and rim hyperenhancement (PHE), so we found persistent THID in 28 (71.8%) lesions (Figure 3).

Control group A: All lesions showed hypointense signals without THID.

Control group B: All lesions showed progressive contrast enhancement.

THID presence, related to the different contrast appearance in this phase, between the groups was statistically different (*p*-value << 0.01 on the chi-squared test).

The portal contrast study pattern showed a difference that was statistically significant in the aspect of lesions among the study and control groups (*p*-value << 0.01 on the chi-squared test).

### 3.5. Equilibrium Phase Appearance

Study group: 32 (82%) lesions showed targetoid appearance and Rim hyperenhancement (PHE), without THID. Seven (18%) lesions showed progressive enhancement.

Control group A: All lesions showed hypointense signal without THID.

Control group B: All lesions showed progressive enhancement.

The equilibrium contrast study pattern showed a statistically significant difference among the groups (*p*-value << 0.01 on the chi-squared test).

## 4. Discussion 

We assessed 17 patients with 39 mucinous subtype metastases (study group), 35 patients with 118 non-mucinous subtype metastases (control group A), and 20 patients with hepatic hemangioma to identify MR features that allow proper lesion characterizations. 

Since the liver is the most common site of distant metastases in patients with colorectal cancer and surgical resection is the only curative treatment [17,18], it is evident that the identification of proper lesions during the pre-surgical imaging setting is the crucial element that should allow an appropriate treatment approach. Moreover, today, it is known that a mucinous subtype lesion has an adverse prognostic impact compared with non-mucinous subtypes, which may influence therapeutic strategies [19]. In this scenario, radiologists should correctly recognize mucinous metastases, which is challenging due to their typical features. To the best of our knowledge, few studies have assessed the radiological features of mucinous colorectal metastases [49,50,51,52]. According to them, mucinous metastases are well-defined nodules with pushing or capsulated growth patterns on histopathology. In our study population, we found pushing or capsulated growth patterns in 25 lesions, while 14 showed an infiltrative pattern, a feature that we found typically in non-mucinous lesions (83 infiltrative patterns vs. 35 pushing or capsulated patterns). In addition, in our study population and study group A, recurrence occurred in 12 patients and only 3 had mucinous previous lesions. These data are different respect to the literature data [19]; however, it could be due to the sample size and the median follow-up time (6 months).

With regard to imaging, the presence of mucin considerably characterizes the lesions’ appearance on MR sequences. In fact, these lesions show hyperintense signals on T2-W and restricted diffusion and iso-hypointense signal on ADC maps. These features could suggest a diagnosis of benign lesions such as hepatic cysts or hemangiomas. According to these results, in our study, we found that all mucinous assessed lesions had a very high SI similar to hemangiomas, while NOS lesions showed a hyperintense signal on T2-W that was less intense than hemangioma. The hyperintense signal and the targetoid appearance on DWI showed a statistically significant difference among the groups (*p* value << 0.01 on the chi-squared test). So as, we found statistically significant difference between the iso-hypointense SI and the hypointense SI on ADC maps among the groups (*p* value << 0.01 on the chi-squared test).

During contrast study, we found that, in arterial phases, all lesions showed Rim APHE, and in the 71.8% of the study group, patients peripheral THID was observed. Conversely, in control group A, only the 21.2% had peripheral THID. Thus, THID presence among the groups was statistically different. The presence of these features (peripheral rim and THID) allows the exclusion of benign lesions, while these features, as reported by Lee et al. [51], have demonstrated a 95% specificity for mucinous metastases. During the portal phase, we found that all mucinous metastases showed non-peripheral washout with targetoid appearance and rim hyperenhancement (PHE), so we found persistent THID in 28 (71.8%) lesions. Instead, in control group A, all lesions showed hypointense SI without THID. TA appearance with persistent rim hyperenhancement has been found also in the equilibrium phase. In seven mucinous lesions, as in hepatic hemangioma, we found progressive enhancement. According to previous studies [49,51], this contrast pattern in mucinous lesions is due to the presence of fibrotic tissues surrounding extracellular mucin pools. Fibrotic tissue presence causes the progressive enhancement from arterial to delayed phases in the contrast study. This appearance and the signal on T2-Wsequences could cause hepatic hemangiomas diagnosis. However, the main significant feature that could help in metastasis diagnosis is the continuous peripheral rim enhancement during the arterial and portal phases [49,50,51]. Recently, there is great attention on radiomics [52,53,54,55,56,57,58,59,60,61,62,63]. Radiomics is a quickly developing field of research concerned with the extraction of quantitative metrics, which include radiomic features, within medical images [54,55,56,57]. Radiomics could support cancer detection, diagnosis, the evaluation of prognosis and response to treatment, and it could supervise disease statuses in oncological patients. Using a standard of care, images are usually obtained in clinical settings. Radiomics analysis is a cost-effective and highly feasible implement for clinical decision support, providing prognostic and/or predictive biomarkers that allows a fast, low-cost and repeatable tool for longitudinal monitoring [58,59,60,61,62,63]. In this scenario, radiomics analysis could help with the identification of mucinous lesions. In our previous study, we demonstrated radiomics data obtained by T2-W sequences in arterial, portal and hepatobiliary contrast phases, and computed tomography (CT) studies allowed the identification of mucinous and NOS lesions [63,64,65,66].

The necessity to recognize mucinous subtype is correlated to the idea that it has a higher risk of metastases, worse overall survival (OS) and an impaired response to conventional chemotherapy [20,21,22]. In our patient population, no statistical differences were found with regard to recurrence, and these data are similar to the results of Reynolds et al., which demonstrated that mucinous CRCLM had similar outcomes to patients with adenocarcinoma NOS [67], so histological subtypes should not be taken into account when deciding on the resectability of CRCLM.

There are several limitations to our study. First, the sample size was small because it is a single-center experience; moreover, the selection of assessed patients, based on patients subjected to surgical resection, could affect the results. Second, this is a retrospective study. Third, we have not assessed radiomics analysis. Our future prospective is to assess radiomics parameters to demonstrate how some parameters could correlate with prognosis and, thus, guide the choice of treatment.

## 5. Conclusions

A proper identification and characterization of liver mucinous metastases allow a better patient selection to avoid unnecessary treatments. 

The diagnosis is challenging since, although the presence of mucin is typical of these lesions, this datum characterizes the appearance of these lesions on MR, which show a high signal on T2-W, restricted diffusion and iso-hypointense signal on ADC maps. However, these features are similar to NOS lesions and hemangioma features. Instead, the continuous peripheral rim enhancement during the arterial and portal phases is typical of mucinous lesions and allows for a proper diagnosis. 

## Figures and Tables

**Table 1:** Characteristics of the study population (52 patients).

Patient Description	Numbers (%)/Range
Gender	Women 19 (36.5%)
	Men 33 (63.4%)
Age	63 years; range: 37–82 years
**Primary cancer site**	
Colon	39 (75%)
Non mucinous type	26 (66.7% of colon cancer patients)
Mucinous type	13 (33.3% of colon cancer patients)
Rectum	13 (25%)
Non mucinous type	9 (69.2% of rectal cancer patients)
Mucinous type	4 (30.8% of rectal cancer patients)
**Hepatic metastases**	
**Non-mucinous type**	35 patients (67.3%) (12 women; 23 men); 118 metastases assessed
**Mucinous type**	17 patients (32.7%) (7 women; 10 men); 39 metastases assessed
Patients with single nodule	22 (64.2%)
Patients with multiple nodules	30(35.8%)/range: 2–14 metastases for mucinous type 2–16 metastases for non-mucinous type
Nodule size (mm)	mean size 36.4 mm; range 7–63 mm
**Growth pattern on histopathology**	
Mucinous type	25 pushing or capsulated 14 infiltrative
Non-mucinous type	35 pushing or capsulated 83 infiltrative
**Recurrence**	12 patients (3 mucinous patients) medium follow-up 6 months
**Control Study Group B**	
Gender	Women 12 (60%)
	Men 8 (40%)
Age	55 years; range: 27–68 years
Hemangioma size (mm)	mean size 25 mm; range 8–43 mm

Imaging Features; The consensus in the assessment of the metastases was 100%. We found no statistically significant differences between the median values of lesion size among the two groups (*p* value > 0.05 for the Kruskal–Wallis test).

## Data Availability

Data are available at https://zenodo.org/record/6450881#.YlhAYOhBy3A (accessed on 25 February 2022).
